# Beyond to the Stable: Role of the Insertion Sequences as Epidemiological Descriptors in *Corynebacterium striatum*

**DOI:** 10.3389/fmicb.2022.806576

**Published:** 2022-01-20

**Authors:** Benjamín Leyton-Carcaman, Michel Abanto

**Affiliations:** Genomics and Bioinformatics Unit, Scientific and Technological Bioresource Nucleus (BIOREN), Universidad de La Frontera, Temuco, Chile

**Keywords:** insertion sequences, epidemiological markers, *Corynebacterium striatum*, emerging pathogen, multi-drug resistance, mobile elements, AMR, machine-learning

## Abstract

In recent years, epidemiological studies of infectious agents have focused mainly on the pathogen and stable components of its genome. The use of these stable components makes it possible to know the evolutionary or epidemiological relationships of the isolates of a particular pathogen. Under this approach, focused on the pathogen, the identification of resistance genes is a complementary stage of a bacterial characterization process or an appendix of its epidemiological characterization, neglecting its genetic components’ acquisition or dispersal mechanisms. Today we know that a large part of antibiotic resistance is associated with mobile elements. *Corynebacterium striatum*, a bacterium from the normal skin microbiota, is also an opportunistic pathogen. In recent years, reports of infections and nosocomial outbreaks caused by antimicrobial multidrug-resistant *C. striatum* strains have been increasing worldwide. Despite the different existing mobile genomic elements, there is evidence that acquired resistance genes are coupled to insertion sequences in *C. striatum*. This perspective article reviews the insertion sequences linked to resistance genes, their relationship to evolutionary lineages, epidemiological characteristics, and the niches the strains inhabit. Finally, we evaluate the potential of the insertion sequences for their application as a descriptor of epidemiological scenarios, allowing us to anticipate the emergence of multidrug-resistant lineages.

## Introduction

Genomic publications in recent years have reinforced the essential role of the niche in the genomic structure of bacteria (Matlock et al., 2021; Shaw et al., 2021). The evolution of bacteria in the different niches is mainly driven by two factors, vertical gene transfer (VGT), and horizontal gene transfer (HGT), due to transduction, transformation, or conjugation events (Lawrence, 2005). The effect of mutations on the evolution of microorganisms is well known, but mobile genetic elements (MGEs) are also important components driving the bacteria evolution. MGEs are the main evolutionary source of pathogenic bacteria in the hospital environment (Djordjevic et al., 2013). However, despite its evolutionary importance, the role of these genomic elements as a whole and their potential use from an epidemiological perspective has been little explored and applied.

Insertion sequences (IS) are small, simple, autonomous, and widely distributed MGE in bacterial genomes. Their size ranges from 700 to 2,500 bp, and they contain a sequence that codes for a transposase flanked by two inversely repeated sequences (IR; Mahillon and Chandler, 1998). Initially, they were considered as “harmful genomic parasites” (Kalia et al., 2004; Vandecraen et al., 2017); however, they are currently considered mutagenic agents that allow the host to adapt to new environmental challenges and colonize new niches (Siguier et al., 2014). In addition, IS are important elements in the formation of other mobile elements such as transposons and plasmids, which is why they are responsible for the mobilization of many genes that confer resistance to antibiotics (ARG) and virulence (Partridge et al., 2018; Razavi et al., 2020).

*Corynebacterium striatum* is a member of the skin and nasal mucosal microbiota in humans; however, its role as an etiological agent of nosocomial and community-acquired diseases is increasingly recurrent due to its multi-drug resistance and biofilm formation capacity (Silva-Santana et al., 2021). Resistance to antibiotics in *C. striatum* has been mainly associated with transposons and plasmids (Olender, 2012) which are part of its resistome, i.e., the total repertoire of genes that contribute to resistance. Recently, the richness of the resistome in *C. striatum* has been evidenced, mainly associated with MGE, where the vast presence of IS in the genomic context of several ARGs was highlighted and underlined the importance of IS in the formation of the resistome in *C. striatum* (Leyton et al., 2021). Whereas insertion sequences have been associated with genes that confer advantageous phenotypes, including resistance genes, studying and relating them could help to better understand the emergence, spread, and persistence of resistance in *C. striatum*, thus making the IS important epidemiological descriptors.

## Why Should Insertion Sequences be an Epidemiological Descriptor?

Classical epidemiology determines the clinical and environmental factors in which disease occurs or relates the routes of transmission and spread of a pathogen. However, with the emergence of molecular epidemiology more than 10 years ago, it has become possible to characterize and determine genetic and environmental factors associated with infectious diseases’ emergence, spread, and pathogenesis. In this context, phylogenetic analyses have made it possible to identify disease lineages and establish the epidemiological links related to infectious diseases. On the other hand, the study of the differential content of genes in strains from different isolation sources has demonstrated the genomic plasticity of these bacteria, suggesting their role in the exploration and establishment of specific niches. Like phylogenetic analyses and differential content analyses, we believe that the IS should be incorporated into the characterization of pathogens such as *C. striatum*, which could help to better understand its pathogenicity, persistence, and transmission dynamics. Here, we explain the reasons why we must consider them epidemiological descriptors.

### Reason 1: *Corynebacterium striatum* Is a Ubiquitous Bacterium

Bacteria ubiquity is explained in part due to their genome size and metabolic capacity (Barberán et al., 2014). *Corynebacterium striatum* has been described as a ubiquitous microorganism; however, the relationships of the adaptative capacity with genomic content are still not entirely clear. The old Baas Becking statement “everything is everywhere, but the environment selects” (O’Malley, 2008) suggests that there are adaptative mechanisms of bacteria related to the niche. *Corynebacterium striatum* has shown a wide range of resistance to antibiotics, and this resistance has probably been obtained and developed in nosocomial settings (Renom et al., 2007; Alibi et al., 2017), leading to the emergence and persistence of antibiotic resistance genes from high their mobility and evolutionary forces (Fondi et al., 2016).

The adaptation and evolution of *C. striatum* can be explained by the insertion sequences. In *Escherichia coli*, it has been suggested that IS could be associated with the genome evolution, fitness, and the formation of new operons (Gaffé et al., 2011; Consuegra et al., 2021; Kanai et al., 2021). This raises the question if the presence of IS would explain the transition of *C. striatum* lineages from normal microbiota to the generation of pathogenic lineages, and whether it would also be related to the role of the environment in the evolution of its pathogenicity. The impact of insertion sequences on the evolution of lineages has been seen in *Shigella* spp., which represents specialized lineages of *E. coli* (Hawkey et al., 2020). Hawkey et al. (2020) pointed out that a part of the historical loss of the metabolic function of *Shigella* spp. is due to the activity of IS and that they continue to play a central role in the ongoing evolution of *Shigella* spp.

Here, we suppose that the IS provide a way of adapting to the niche in *C. striatum*. It has been suggested that IS abundance is positively related to genome size and HGT events (Touchon and Rocha, 2007). Moreover, through “transposition bursts,” the IS arise in environmental stress; IS can persist or become extinct in the host, depending on the adaptive regulation that the host possesses (Wu et al., 2015). This suggests that the dynamics of the IS are variable, and they can provide information on the evolutionary state of bacteria like *C. striatum*. In this perspective, we propose that the evolution of *C. striatum* into opportunistic states or pathogenic lineages is the product of the selective pressure of the environment, reflecting mainly the presence and abundance of IS. Therefore, we suggest the role of the IS as epidemiological descriptors in *C. striatum*.

### Reason 2: The Presence of Plasmids in *Corynebacterium striatum* Is Rare

Historically, the pathogenicity of bacteria has been associated with large MGEs, such as bacteriophages and plasmids. It is known that plasmids are increasingly persistent in a bacterial community (Alonso-del Valle et al., 2021), and there are approaches such as plasmid classification and typing proposed to monitor antibiotic resistance (Orlek et al., 2017). Yet despite the approaches to applying plasmids as surveillance in the epidemiology of certain bacteria such as *Klebsiella pneumoniae* (Ramirez et al., 2019), in *C. striatum* seems to be difficult due to difficulties in determining the presence of plasmids. As of the writing of this manuscript, four plasmids have been registered in *C. striatum* (pTP10: NC_004939.1, FDAARGOS_1197 plasmid unnamed: CP069515.1, pCs-Na-1: CP021253.1, and pCs-Na-2: NZ_CP021254.1). The plasmid pTP10 is the best known: it was reported in *C. striatum* M82B, a strain dated approximately 1983 (Kono et al., 1983; Tauch et al., 2000). However, there are no studies related to the other three plasmids. Although there have been attempts to find plasmids (Ramos et al., 2018), to date, no other plasmids have been reported (08-12-2021), probably because they do not exist or because the pTP10 or related plasmids have been integrated into the genome of *C. striatum*. This latter idea is reinforced by two of the four known plasmids corresponding to strains from approximately 40 years ago, whereas the other two plasmids correspond to a strain isolated from *Neophocaena asiaeorientalis*, a fresh water cetacean, and which represents a very different environment from the nosocomial setting.

The plasmids in bacteria provide a positive platform to capture and spread a variety of ARG. In corynebacteria of clinical importance, several plasmids are known to confer resistance (Deb and Nath, 1999; Leyton et al., 2021), such as pTP10 of *C. striatum*, pNG2 of *Corynebacterium diphtheriae*, and pJA144188 of *Corynebacterium resistens*. They also include a significant content of insertion sequences (Tauch et al., 2003; Schröder et al., 2012), suggesting a relation between IS, plasmids, and *C. striatum*. Furthermore, this extends to other plasmids and other bacteria; for example, Che et al. (2021) showed that IS contribute to the acquisition and transfer of ARG in conjugative plasmids. Regardless of the existence of plasmids, something that is quite clear is the relationship of IS with antibiotic resistance in *C. striatum* (Wang et al., 2020; Leyton et al., 2021). Therefore, the increase in resistance in *C. striatum* strains would be mainly associated with these mobile elements.

### Reason 3: Analysis of the Content of Insertion Sequences in *Corynebacterium striatum* Isolates Shows Relationships With Epidemiological Factors

**Figure 1:**
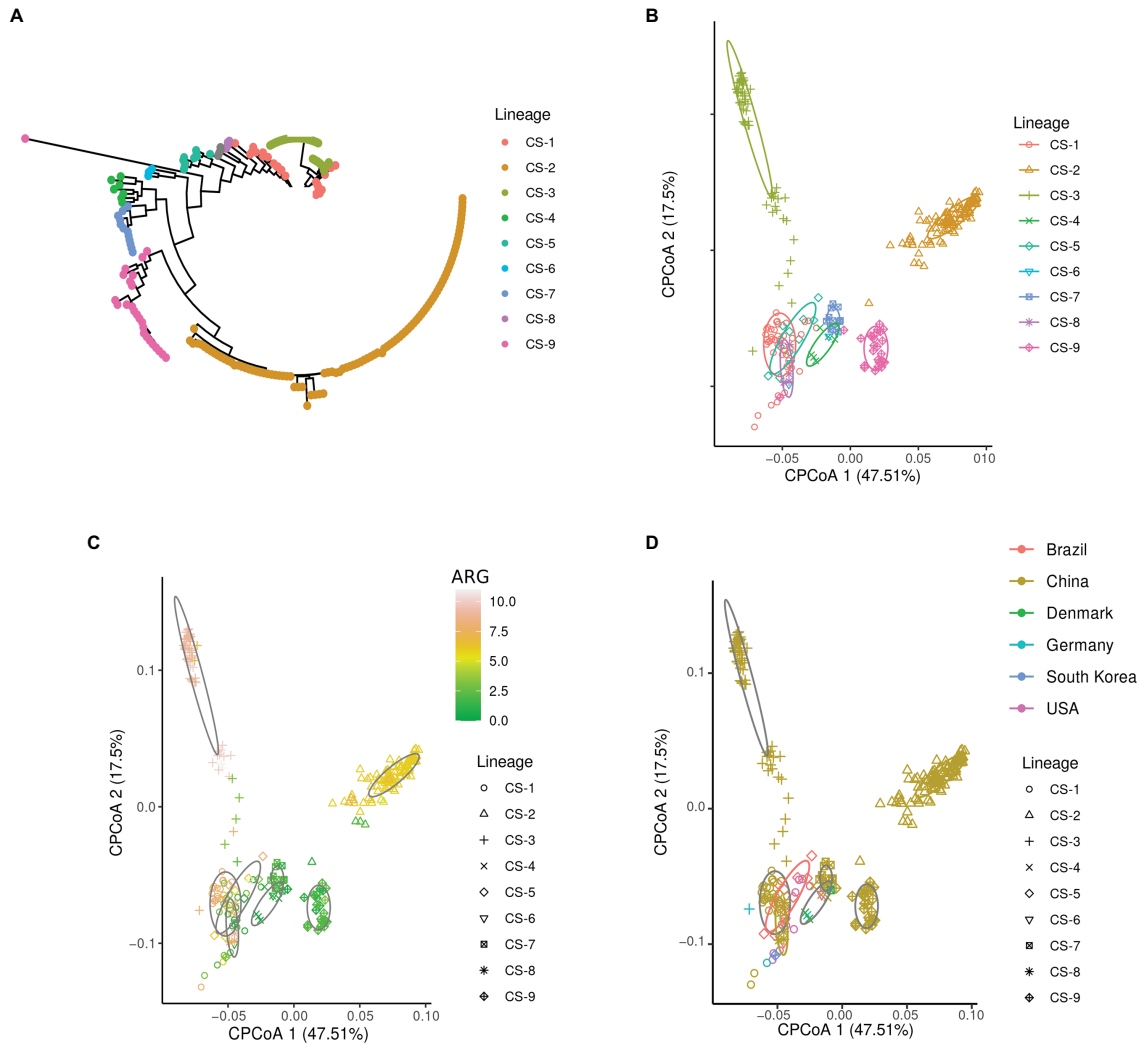
Relationship of IS with epidemiological characteristics. **(A)** Lineages of *Corynebacterium striatum*. **(B–D)** Constrained principal coordinate analysis (CPCoA analysis). Lineages were determined using the R package, RhierBAPS (Tonkin-Hill et al., 2018) using a masked alignment generated by Gubbins (Croucher et al., 2015), and phylogenetic tree generated by IQ-TREE (Nguyen et al., 2015). The CPCoA analyses (ANOVA-like permutation analysis, value of *p* < 0.0004, and 24.9% of variance) were constructed from the presence and abundance of IS in the *C. striatum* genomes and explored according to the metadata available and retrieved from different sources in this study. Briefly, we obtained the *C. striatum* genomes available at NCBI (07/26/2021), made the annotation with Prokka (Seemann, 2014) and constructed its pangenome with Panaroo (Tonkin-Hill et al., 2020). The presence and abundance of insertion sequences were extracted from the pangenome. For phylogenetic reconstruction, we align the core-genome using Parsnp (Treangen et al., 2014), and we build a tree based on core-genome alignment using IQ-TREE. The presence of resistance genes was extracted from the metadata available from the NCBI, a database that annotates the genomes using AMRFinderPlus (Feldgarden et al., 2021). Finally, the visualization of the CPCoA analyzes was used BIC (http://www.ehbio.com/Cloud_Platform/front/#).

To determine the diversity of the insertion sequences in *C. striatum*, we recovered 268 genomes from *C. striatum*, identified the lineages, and looked for clustering patterns using constrained principal coordinate analysis (CPCoA) from a large dataset (Supplementary Data 1). Despite the definition of IS, to perform the analyses in this study, we defined the IS in terms of their transposase. This dataset includes the presence and abundance of insertion sequences detected by Prokka (Seemann, 2014) and grouped by Panaroo (Tonkin-Hill et al., 2020). The details and code used for this methodological approach are available on GitHub.^1^ We found a relationship between epidemiological factors and the composition of insertion sequences (Figure 1; ANOVA-like permutation analysis, value of *p* < 0.0004). Thus, we found an association between lineages and IS composition (Figure 1B); this suggests an adaptation and a population cohesion around the content and abundance of IS. Subsequently, we evaluated the abundance of ARGs in the lineages and IS patterns. Interestingly, we found lineages with a more significant number of ARGs (Figure 1C). Moreover, these lineages, including CS-2 and CS-3, are phylogenetically distant from the rest, indicating a specialization of the lineages according to their IS content.

**Figure 2:**
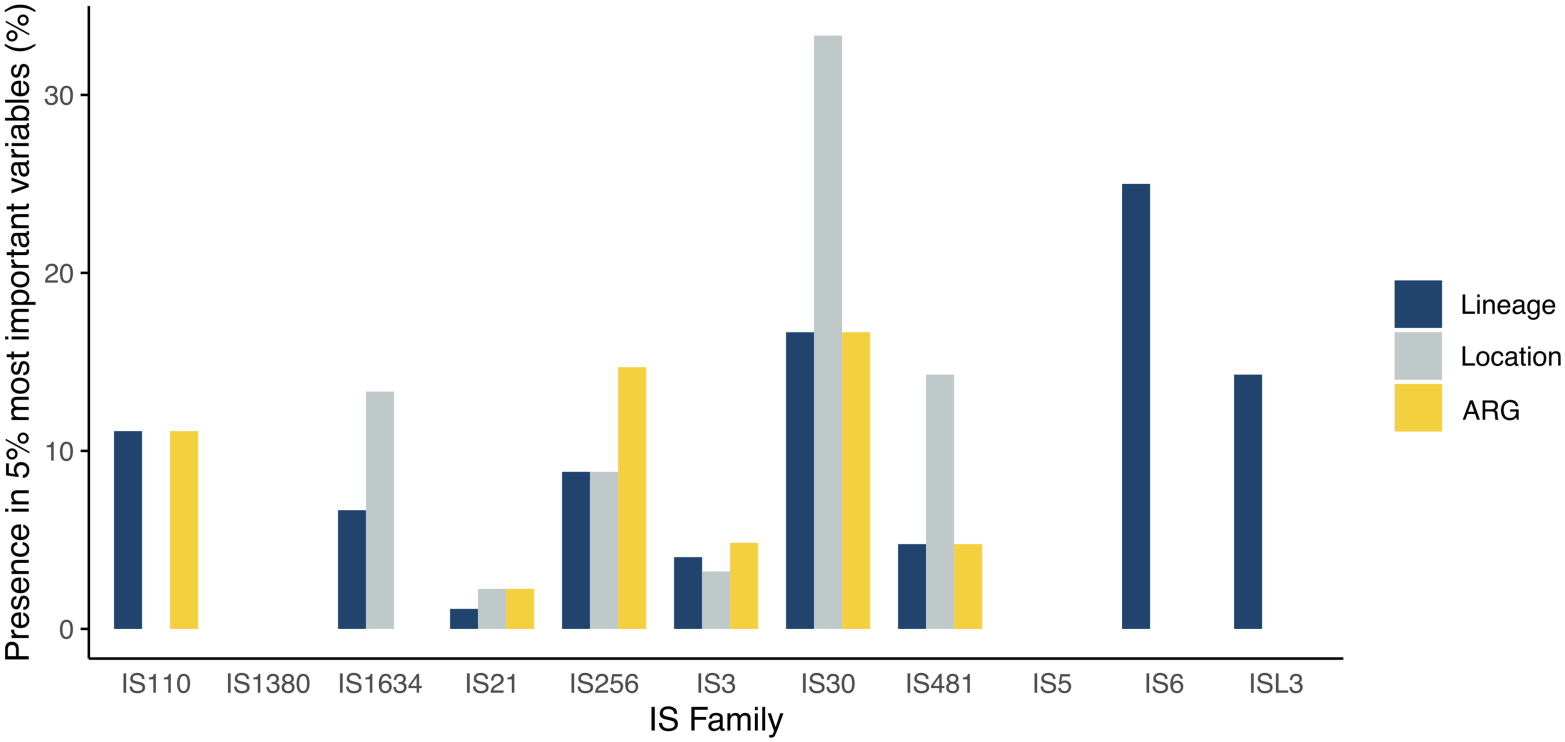
The importance of IS Families in each epidemiological variable. Presence of 5% of the most important functionally annotated insertion sequences for Random Forest (RF) in each epidemiological variable evaluated. All RFs consisted of 501 trees each and were calculated using the random forest 4.6-14 package (Breiman, 2001; default parameters) in version R 4.1.0 (The R Project for Statistical Computing; http://www.r-project.org). For RFs validation, a *k*-Fold Cross Validation test (*k*-fold = 100), and a Kappa concordance test were carried out (for lineage classification, *k* = 0.963, value of *p* < 0.0001; for Location classification, *k* = 0.597, value of *p* < 0.0001; for ARG classification, *k* = 0.786, value of *p* < 0.0001). Finally, the importance of each variable was based on the mean decrease Gini index respecting the pathline described by Dutilh et al. (2014).

To determine the importance of the IS content in epidemiological characteristics analyzed in this study, we performed a random forest (RF) analysis. Since its development in 2001, RF has become a popular tool in omics research (Dutilh et al., 2014). RF has the advantage of incorporating a vast and diverse amount of data to predict characteristics. We consider that RF can provide a potential application to classify epidemiological features in *C. striatum*. IS, being abundant and diverse mobile elements, become difficult to analyze. Thus, we built a RF model for each characteristic evaluated in this study (Figure 2). We found that there are families (IS6 and ISL3) associated with the lineage. In addition, we found that the IS corresponding to the IS30, IS481, IS3, IS21, and IS256 families have great importance in the classification in the three epidemiological categories studied. In addition, IS380 and IS5 are not important for the RF classification. Interestingly, a member of the IS30 family associated with daptomycin resistance was recently reported (Gotoh et al., 2021), which together with this perspective highlights the importance of IS as evolutionary triggers in *C. striatum*.

## Discussion

The use of insertion sequences is not new in bacterial pathogen studies, having been used as epidemiological markers in some species. A specific example is IS6110 in the genus *Mycobacterium*, where this IS has been used as a typing tool. The number of copies of this element would be related to the species identification and the strain typing, demonstrating an essential value in the epidemiology of *Mycobacterium* spp. (van Soolingen et al., 1991; Suffys et al., 1997; Roychowdhury et al., 2015). We propose that the importance of insertion sequences lies in describing the environment or niche in which the pathogen is found. In a nosocomial environment, MGEs play a fundamental role in the emergence of pathogens due to genes that confer resistance to antibiotics or virulence and, therefore, with differing levels of fitness. Several studies have identified the presence of IS and addressed the role of larger genomic elements, such as plasmids and integrons, in the epidemiology of some pathogens (Rozwandowicz et al., 2018; Ramirez et al., 2019; Hawkey et al., 2020; Leal et al., 2020; Mbelle et al., 2020). Recently, a high presence of IS has been evidenced, composing conjugative plasmids, and that would be overrepresented in the presence of ARG (Che et al., 2021). We propose that attention focus on IS assumed to be mobile functional monomers capable of being part of larger mobile elements. Furthermore, we think that IS could be an indicator of risk or alarm because the presence of IS could provide information on the pathogenic evolution of the bacteria that inhabit the nosocomial niche.

Based on our results, the IS profile could be a framework for assessing the health risk of antimicrobial resistance genes. The diversity and abundance of ARGs associated with IS, allowed us to infer high-risk lineages such as the CS-2 and CS-3 lineages compared to other lower risk lineages due to having fewer ARGs. According to the analyzed data, most of the available sequences of *C. striatum* are from China (Figure 1D), and a recent epidemiology study found that isolates from various cities in China were separated into four lineages, which would have originated more than 20 years ago (Wang et al., 2020). Interestingly, the IS patterns generated in our analysis identified that the Chinese isolates were separated into six clusters, of which CS-1, CS-2, CS-3, and CS-9 would be more specialized in terms of IS. Moreover, our results suggest that the IS profile allows a fine-tuned lineage characterization related to the presence of a more significant number of ARGs.

In this study, 16,360 copies of IS were found, grouped into 323 clusters of gene families. From the results obtained, we found that the abundance of IS could explain the capacity of *C. striatum* to acquire antibiotic resistance. Yet a high abundance of IS not strictly synonymous with HGT (Touchon and Rocha, 2007); rather, a high transposition of IS tends to be detrimental to the host (Wu et al., 2015). This does not seem to be the case for *C. striatum*, however, which suggests there must be regulation mechanisms in *C. striatum* to obtain the persistence of the IS and of the bacterium itself. To explore whether the transposition of the IS has been affecting the evolution of *C. striatum*, we explored the dynamics of recombination and mutation of the genomes analyzed using ClonalFrameML (Didelot and Wilson, 2015). We estimated the relative impact of recombination around four times more than to mutation in genomic diversification (*r*/*m* = 4.4), suggesting a clear role of homologous recombination events in genomic plasticity and evolution in *C. striatum*. Strictly, not all recombination events can be attributed to the IS activity; however, in the absence of other mobile elements, the IS could play an important role in *C. striatum*. For example, the *r*/*m* value of *C. striatum* is lower than that reported in a *C. diphtheriae* population (*r*/*m* = 5; Hennart et al., 2020). Moreover, the recombination of *C. diphtheriae* is influenced by other larger MGEs such as plasmids and integrons (Tauch et al., 2003; Barraud et al., 2011; Hennart et al., 2020).

Genetic determinism has tended to follow the simplistic idea that single genes control a disease (Ahn et al., 2006; Loscalzo et al., 2007; Williams and Auwerx, 2015). Based on this assumption, it has probably led us to look for single gene markers, and why IS as unique genes and markers have not generated much interest. However, considering the current microbial genomics scenario where we can now obtain whole-genome information, we propose that the study of the IS composition should be used as an epidemiological descriptor of *C. striatum*.

### Limitations and Future Approaches

In addition to what we have explored in this study, another important point to be explored would be the association of IS with genes in their genomic context in order to achieve a greater understanding of the functional role of IS. Nevertheless, due to the structure and genomic context of IS, their characterization and their genomic context could be affected by short-read sequencing technologies that produce fragmented genome assemblies, which could be a limitation for the recovery of IS and the characterization of their genomic context. To overcome this limitation, the use of long-read sequencing technologies such as nanopore or PacBio, alone or in combination with short-reads technologies, could help produce complete genomic assemblies and thus perform a better characterization of ISs. In addition, it is crucial to evaluate the entire repertoire of ISs, which would most convincingly explain the niche or the environments that host them.

Our study based on IS composition identified genomes clustered according to the phylogenetic lineages, a potential application of insertion sequences as epidemiological descriptors. To study a large number of genes to describe epidemiological features could be a hard task to undertake. Implementation of integrative approaches such as machine learning and deep learning in microbiology and genomics has gradually increased due to the large amounts of data generated by high-throughput sequencing technology (Libbrecht and Noble, 2015; Qu et al., 2019). Our analyses show specific patterns of the presence and abundance of IS with the detected lineages or related to ARG composition. Classification techniques such as Random Forest (as described in this study) and Super Vector Machine (SVG) could be valuable for classifying epidemiological features related to *C. striatum* based on its IS composition. Moreover, unsupervised machine-learning techniques could help find new IS patterns in *C. striatum*.

Currently, there is an inclination to “dissect” deep learning models (Bau et al., 2017). Unveiling neural layer learning, a concept is known as “opening the black-box,” consists of obtaining a better interpretation of how deep learning models work (Shwartz-Ziv and Tishby, 2017). In this sense, a future application would be to train the IS patterns by deep learning and then dissect these models to identify the role of certain IS in different epidemiological scenarios of *C. striatum*.

On the other hand, microbial genome-wide association studies (mGWAS) are a new field that seeks to understand how variations in microbial genomes affect the phenotype of a pathogen, such as drug resistance and virulence (Power et al., 2017; San et al., 2020). mGWAS bioinformatics tools use machine learning intrinsically in their algorithms, such as PySEER and Kover, for phenotype prediction (San et al., 2020). Therefore, mGWAS becomes a promising alternative for studying insertion sequences and their relationship with phenotypes in *C. striatum*. All these approaches must be considered due to the currently available genomic data, which can be a way forward to understanding the epidemiology and evolution of emerging pathogens such as *C. striatum*.

In conclusion, taken together, we propose the use of the IS composition as epidemiological descriptors, i.e., as a set of genomic data that can describe epidemiological features. Descriptions could include the transient or persistent state of *C. striatum*, the relationship with the lineage to which they belong (and the role of the IS in the establishment of these lineages in the face of the selective pressures of the environment); as well its use in surveillance and emergence of new lineages. Finally, the study of the composition of ISs through exploratory and integrative big-data approaches could facilitate the ecological and evolutionary understanding of antibiotic resistance in *C. striatum*.

## Data Availability Statement

The original contributions presented in the study are included in the article/Supplementary Material, further inquiries can be directed to the corresponding author.

## Author Contributions

BL-C performed the data curation, formal analysis, investigation, visualization, and writing the original draft. MA performed the conceptualization of the study, investigation, supervision, and writing – review and editing of the manuscript. All authors contributed to the article and approved the submitted version.

## Conflict of Interest

The authors declare that the research was conducted in the absence of any commercial or financial relationships that could be construed as a potential conflict of interest.

## Publisher’s Note

All claims expressed in this article are solely those of the authors and do not necessarily represent those of their affiliated organizations, or those of the publisher, the editors and the reviewers. Any product that may be evaluated in this article, or claim that may be made by its manufacturer, is not guaranteed or endorsed by the publisher.

## Footnotes

^1^ https://github.com/Leytoncito/IS_Prokka_Panaroo

## Supplementary Material

Supplementary Data 1 Data collected from NCBI and data generated in this study. This material contains the list of genomes studies, annotation of insertion sequences, and a matrix with the presence and abundance of insertion sequences per genome.
